# The ctDNA revolution: Insights on cancer care: A narrative review

**DOI:** 10.6026/9732063002001287

**Published:** 2024-10-31

**Authors:** Krutika Mahendra Gohil, Jyothika Venkataswamy Reddy, Saniya Kifayetulla Mulla, Rutu Snehal Shah, Shrunga Kandhi Raghavendra, Rishika Singh, Sagar Mahendra Gohil

**Affiliations:** 1 Department of Internal Medicine, Hinduhridaysamrat Balasaheb Thackarey Medical College (HBTMC) and Dr. Rustom Narsi Cooper Municipal General Hospital, Mumbai, Maharashtra, India; 2 Department of Medicine, Chamarajanagar Institute of Medical Sciences, Chamarajanagar, Karnataka, India; 3 Department of Medicine Dr. Vaishampayan Memorial Government Medical College, Solapur, Maharashtra, India; 4 Department of Medicine Mahatma Gandhi Mission Institute of Health Sciences, Navi Mumbai, Maharashtra, India

**Keywords:** ctDNA, tumor specific DNA, cancer, predictive marker, genomics

## Abstract

Circulating tumor DNA (ctDNA) is a revolutionary tool in the detection and monitoring of cancer: a minimally invasive, highly sensitive approach to analysing tumor-specific DNA in the bloodstream. Therefore, it is of interest to explore the current and evolving landscape of cancer genomics as precision tools in quantifying tumor dynamics. Thus, the role of ctDNA in tracking minimum residual disease, relapse, recurrence and the tailoring of therapeutic strategies for effective management of tumours is reviewed.

## Background:

Circulating tumor DNA (ctDNA) is fragmented DNA which cancer cells release into the bloodstream [1]. CtDNA is a non-invasive method for assessing the kinetics and genetic changes of a tumor [2]. Recent research indicates that it is also useful for screening cancer during its early stages [3]. It detects genetic changes and mutations that often precede clinical use of standard imaging methods [4]. Besides these, the prospect of ctDNA as a predictive marker for minimal residual disease and assessment of therapy responses has also been promising [5]. Nevertheless, despite these promises, various aspects like sensitivity, specificity and standardization of assays still need to be addressed before incorporating the use of ctDNA in clinical practice [2]. The present review encompasses the current scenario of the application of ctDNA in diagnosing, monitoring and treatment of cancer.

## Biological characteristics of ctDNA:

## Origin of ctDNA in the blood:

ctDNA is released by tumor cells through apoptosis, necrosis, or active secretion [6, 7]. It can be released by both primary and metastatic tumors, which makes it an important marker for detecting various cancer types [8, 9].

## Structural and molecular features of ctDNA:

ctDNA is composed of small pieces similar to the dimensions of apoptotic cfDNA pieces. Generally, it is around 150 to 200 base pairs in length [10]. It is different from cfDNA because it holds tumor-specific changes like point mutations, chromosomal rearrangements, copy number variations, and DNA methylation profiles [11]. All these characteristics of ctDNA are manifestations of the root genomic instability of the tumor [12].

## Determinants of blood ctDNA concentrations:

Several factors have been shown to affect the ctDNA levels in blood, including the size, type, location, and cell turnover rate of the tumor [13]. In addition, more progressed tumors often have higher levels of ctDNA concentrations depending on the stage of the tumor and the existence of metastases [14, 15].

## Detection methods of ctDNA:

## Digital polymerase chain reaction droplet (DDPCR):

This is one of the best methods for analyzing genomic changes that exist, in terms of use and accuracy. This involves the use of a water-oil emulsion droplet technology, which creates hundreds to millions of droplets within the DNA sample itself and forms the basis of this technique. Because fluorescent TaqMan-based probes can be distinguished with just one mutant or non-mutated DNA strand within a droplet, it detects them through flow cytometry [17].

## Beads, emulsion, amplification and magnetic beaming:

Beaming is one of the methods relatively cheaply and sensitive to screen for a few possible mutations [16]. This technique links the amplified target DNA fragment to magnetic beads after amplification by primers with known tag sequences. In the end, the beads carrying the mutation are sorted with flow cytometry [18].

## Cancer personalised profiling by deep sequencing (CAPP-Seq):

More thorough diagnostic data can be obtained by using CAPP-Seq, which can detect many mutations in patients with the same kind of cancer and enhance the evaluation of tumor heterogeneity [16]. All major mutation types, such as insertions, rearrangements, single nucleotide variations, and copy number changes, can be found using CAPP-Seq [19].

## Mass spectrometry:

The mass spectrometry-based approach includes a traditional multiplex PCR in addition to matrix-assisted laser desorption/ionisation time-of-flight mass spectrometry [20].

Surface-enhanced Raman Spectroscopy (SERS) is used in the SERS-PCR detection method to identify and track the binding target utilising nano tags, which are nano particulate optical detection tags [17]. Two-step PCR is used in Ultra SEEK for amplification, while mass spectrometry is used for detection. The two-step PCR process comprises of a single-base extension reaction that is unique to a mutation, followed by a multiplex PCR [20].

## Clinical applications of ctDNA:

## Early detection and screening of cancer:

Compared to the previous plasma biomarkers, ctDNA is superior in two key areas: sensitivity and clinical affiliation [18]. Connectedly, Bettegowda *et al.* conducted another study exploring the ctDNA horizon, which also demonstrated that ctDNA was more sensitive than both protein biomarkers and CTC in cancer diagnosis [21].

## Monitoring disease progression, treatment response and relapse status:

ctDNA precisely conform the real-time tumor burden in patients undergoing cancer treatments. Phallen *et al.* (2019) conducted a study of 31 patients with stage I-IV colorectal cancer who underwent potentially curative surgeries. The subsequent findings related higher levels of ctDNA with worse outcomes [22]. Similarly, Diehl *et al.* analyzed that most patients experienced a significant reduction or complete absence of ctDNA levels following surgery. Subsequent workup revealed that post-surgery ctDNA tracings pointed towards relapses versus remission in patients with no detectable ctDNA [23]. In addition to determining treatment responses, ctDNA possesses the ability to predict cancer relapses months before they clinically emerge. This allows for timely intervention and to combat cancer before it recurs or metastasizes. The study by Garcia-Murillas *et al.* confirms the relapse-predicting capability of ctDNA [24].

## Detection of minimal residual disease (MRD):

In a notable portion of cancer patients, small numbers of remaining tumor cells, called minimal residual disease, can persist at levels too low to be detected by imaging or physical examinations, potentially resulting in disease relapse. Increasing data shows that ctDNA as a marker of MRD after treatment for solid tumors can predict relapse. This could help recognize patients who might benefit from adjuvant therapy and assess their response to such treatment [25].

## ctDNA in personalized medicine and targeted therapies:

The qualitative aspects of ctDNA have been found useful in designing targeted cancer therapies for patients based on distinct mutations occurring on ctDNA [26]. An instance is- the presence of mutant PIK3CA in tissue or ctDNA of patients with hormone receptor-positive, HER2-negative metastatic breast cancer allows the use of the PI3Kα inhibitor alpelisib [27].

## Role of ctDNA in different types of cancer:

## Breast cancer:

Dawson *et al.* investigated ctDNA, circulating tumor cells (CTC) and CA15-3 in 30 patients with metastatic breast cancer and found that ctDNA was detected in 97% of cases, whereas CTC and CA15-3 had detection rates of only 78% and 87%, respectively [28]. Ma *et al.* showed that ctDNA genotyping is a specific and efficient way to track resistance to targeted treatments and identify novel resistance pathways in MBC that were HER2-positive. Furthermore, ctDNA sequencing is used to pinpoint new resistance mechanisms. With a concordance rate of up to 82.1%, dynamic ctDNA profiling using pre-set criteria was more sensitive than CT scans in diagnosing drug resistance [29].

## Metastatic melanoma:

Recent research indicates that ctDNA analysis is a useful tool for melanoma diagnosis and prognosis. CtDNA can be used to provide a real-time assessment of patient status and, in cases of emerging relapse, it can act as a trigger for adapting treatment strategies in response to the biological changes detected. This is because the goal of therapy in metastatic melanoma is shifting from disease stabilization to longitudinal complete response [30]. The levels of ctDNA were found to have a significant correlation with clinical serological markers of disease burden, such as lactate dehydrogenase (LDH), S100 calcium-binding protein B (S100B) and melanoma inhibitory activity (MIA). However, ctDNA was shown to be a more precise indicator due to its tumor-specific origin and its capacity to cover a dynamic range of 4-5 log scale units [31].

## Pancreatic cancer:

The circulating tumor DNA is used in evaluating prognosis, screening, relapse, targetable mutations and resistance mutations in pancreatic cancer. Lee *et al.* indicated ctDNA is detectable at diagnosis but becomes undetectable post-operatively and that this is associated with a reduction in relapse risk compared with those where ctDNA remains detectable [32].

## Liver cancer:

Ono *et al.* highlighted the value of detecting ctDNA for tailoring treatment in liver cancer [33]. In a prospective study of 26 pancreatobiliary malignancies, Zill *et al.* found a high agreement between mutations identified in tumor biopsies and cfDNA. This study established that ctDNA could address tumor heterogeneity, tumor burden and prognosis [34]. Furthermore, another study demonstrated that ctDNA could track therapeutic responses in real-time and manage longitudinal monitoring effectively [35].

## Non-small cell lung cancer:

The TRACERx research is the first prospective investigation on post-lung cancer surgery ctDNA changes. It tracked clonal changes in 100 patients with early-stage NSCLC using tumor-specific phylogenetic approaches. With a median lead time of 70 days before imaging confirmation, 93% of the 14 patients who experienced recurrence had detectable SNVs either before or at the recurrence [36]. Further research increased the median lead time to 151 days by discovering ctDNA in 77% of recurrent instances. With a median lead time of 5.2 months, ctDNA detected Minimum Residual Disease (MRD) in 72% of patients prior to imaging in other studies utilizing CAPP-Seq, allowing for earlier clinical intervention [37]. Furthermore, ctDNA's potential as a predictive biomarker was demonstrated by the correlation between ctDNA clearance and longer progression-free survival in EGFR-positive patients receiving targeted therapy [38].

## Early-stage colon cancer:

Most early-stage colon cancer patients are cured by surgery alone; the 5-year disease-free survival (DFS) rate for low-risk stage III patients is 78%, and for stage II patients it is 78-91% [39]. However, because there are no trustworthy biomarkers for minimal residual disease (MRD), adjuvant chemotherapy (ACT) is advised for all stage III patients. A study by Tarazona *et al.* evaluated 150 resected colon cancer patients using serial ctDNA testing, showing a strong correlation between postoperative ctDNA detection and relapse. Combining ctDNA with serum CEA improved sensitivity from 58.8% to 63.6% [40].

## Limitations and future perspectives:

In light of the challenges, future perspectives for ctDNA testing involve the need for well-designed clinical trials to establish it as a promising biomarker. It is crucial to address early cancer detection limitations and consider its clinical usefulness as an alternative to tissue biopsy for challenging-to-diagnose tumors. Furthermore, exploring the complementary use of circulating tumor cells (CTCs) and ctDNA for monitoring tumor changes and treatment response holds promise for advancing cancer care [20]. Our approach opens opportunities for the discovery of additional multi-feature genomic predictors coming from ctDNA in various cancer-types as also shown by different previous studies [41- 42].

## Conclusion:

The circulating tumor DNA (ctDNA) stands out as a transformative innovation in oncology, offering a non-invasive approach to track tumor behaviour, assess treatment efficacy and monitor disease progression. Its ability to provide real-time insights into the genetic landscape of tumors positions ctDNA as a crucial component of personalized cancer care. As research advances, ctDNA has the potential to revolutionize early detection, guide therapeutic decisions and ultimately enhance patient outcomes. The continued development and integration of ctDNA testing into clinical practice will be essential in shaping the future of cancer management.
